# Characteristics of workplace violence, responses and their relationship with the professional identity among nursing students in China: a multicenter cross-sectional study

**DOI:** 10.1186/s12912-022-01037-3

**Published:** 2022-09-26

**Authors:** Lingyan Zhu, Lu Dongyan, Zhenlan Luo, Xu Mengqi, Linfang Sun, Hu Sanlian

**Affiliations:** 1 Department of Nursing, Shanghai JiaoTong University Affiliated Sixth People’s Hospital, Shanghai, China; 2 School of Nursing, Huzhou University, Huzhou, China; 3 School of Nursing, Shanghai JiaoTong University School of Medicine, Shanghai, China

**Keywords:** Workplace violence, Professional identity, Nursing students, Clinical practice

## Abstract

**Background:**

Nursing students are one of the most vulnerable groups suffering from workplace violence. This study aimed to investigate the workplace violence incidence of Chinese nursing students during clinical practice, to describe the characteristics of violence and students’ responses and to analyse the relationship between the experience of workplace violence and professional identity.

**Methods:**

A cross-sectional study was conducted among 954 nursing students in ten universities in China through convenience sampling. Workplace violence was surveyed through Hospital Workplace Violence Questionnaire for Nursing Students. Students’ professional identity was measured by Professional Identity Questionnaire for Nursing Students. Statistical methods included descriptive statistics, chi-square test, independent-samples t-test, analysis of variance and linear regression analysis.

**Results:**

It was found that the incidence of workplace violence among nursing students was 42.98%. The violent incidents ranking from high to low were: verbal abuse, threat, physical attack, sexual harassment, and gathering disturbance. The majority of the nursing students chose to avoid the conflict during the incident. 86.34% of the students didn’t report the incidents. More than half of the victims developed post-traumatic stress disorder after the incidents. Regression analysis results showed that workplace violence experience (β = − 0.076, *P* < 0.05) was a significantly negative predictor of professional identity.

**Conclusions:**

Chinese nursing students were exposed to physical and psychological violence during clinical practice with insufficient coping solutions and psychological adjustment. The professional identity of the nursing students was significantly associated with the experience of workplace violence.

## Introduction

Workplace violence (WPV) is defined as ‘Incidents where staff is abused, threatened or assaulted in circumstances related to their work, including commuting to and from work, involving an explicit or implicit challenge to their safety, well-being or health’, including physical and psychological violence [1]. WPV was reported occurring in 68.31% nursing staff, including 25.77% physical WPV and 63.65% non-physical WPV [2]. Meanwhile, nursing student is one of the most vulnerable and high-risk group suffering WPV, due to inadequate experience in clinical practice, frequent clinical placement shifts and challenges of building the relationships with patients and multidiscipline team in a short period [3]. Recent studies revealed that more than half of the nursing students experienced WPV during clinical practice [4–6]. Wang [6] reported 66.2% Chinese nursing students suffered from WPV, including emotional abuse (77.6%), threat (55.9%), physical aggression (15.2%) and sex assault (10.3%). 84.8% of violence was identified by patients and patients’ family members, followed by nurses (78.6%) and physicians (57.9%) [7, 8]. Another study revealed most WPV came from patients and relatives (77.1%) [9]. Magnavita and Heponiemi [10] found that psychiatric and emergency departments were the highest risk work environments. However, the characteristics of violence and the responses of nursing students were unclear. Research found that midwifery students discussed the incident with acquaintance after a violence incident and few of them completed an incident report or received official debriefing [11]. Some students may worry about losing their job after the violence, while others do not trust the hospital staff.

WPV was considered causing long-term physical and psychological impact to the nursing students [12]. A survey in China indicated that 59.1% of the nursing students worried about WPV in the clinical practice [13] and the majority of the students experiencing WPV showed anxiety and depression [5]. Nursing students who witnessed or experienced WPV reported more psychological problems such as fear, anger and irritation than others [3]. WPV also impacts the students’ nursing performance. 12.3% of students announced that WPV experience influenced the quality of patient care [14]. Additionally, WPV deteriorated the professional acceptance of nursing students [1]. Budden et al. [5] found that 46.9% of the nursing students considered to change their careers after experiencing WPV. The students received inadequate WPV management training during the nursing education. It is necessary to clarify the impact of WPV to build up the professional identity among the nursing students, who are considered to be primary backup forces of the professional nurses.

Although many universities and hospitals tried to change the culture of clinical practice in China, the evidence of the change was still unclear. The objectives of this study are: (i) to analyse the violence incidence and experience among Chinese nursing students during clinical practice; (ii) to investigate the relationship with the development of students’ professional identity.

## Methods

### Design

A cross-sectional study design was used following the Strengthening the Reporting of Observational Studies in Epidemiology (STROBE) guidelines [15].

### Participants

Nursing students from 10 universities/colleges in 5 cities in China: Shanghai, Suzhou, Dali, Nanchang, and Hefei were recruited to this study by convenience sampling method from March 1st 2019 to May 31st 2020. We calculated the sample size as per the following formula:

$$\mathrm{n}=\frac{{\upmu^2}_{\upalpha /2}\kern0.1em \uppi \left(1\hbox{-} \uppi \right)}{\updelta^2}$$

(where α = 0.05，μ_α/2_ = 1.96，δ = 0.05). In order to ensure sufficient sample size, π = 50% was set for calculation [16]. Considering 20% non-response rates, we found the minimum sample size was 461. Since this was a multi-center survey, we tried to collect as many sample sizes as possible to improve the sample representatives. The entry criteria are: ①The nursing students in the final year of clinical practice, ②Students consented to participate in this study. Exclusion criteria: student whose sick leave was longer than 1 month.

Ethical approval was obtained from Huzhou University Research Ethics Committees (20190910). The questionnaires were issued to the nursing students in an internship after they returned to school for academic study. All students were required to sign a consent form after the aim and method of this study were explained to them. The questionnaires were filled anonymously and should be completed in 10 to 15 minutes and collected in-situ. 1094 nursing students were recruited in this study and 954 questionnaires (87.20%) were completed.

### Instruments

A General Information Survey Sheet was designed including gender, age, family residence, education background, major selection incentives, training of violence management, and the concern about violence.

The Hospital Workplace Violence Questionnaire, which was designed by Chen and revised by Yang, was used widely in China [17, 18]. The Cronbach’s α of the questionnaire was 0.755. In this study, the questionnaire was revised according to the study purpose and characteristics of the nursing students and renamed as Hospital Workplace Violence Questionnaire for Nursing Students (HWVQNS). The HWVQNS is composed of two parts: various types of violence occurred in the internship and a description of a violence incident (including the place of the violence, the perpetrator, the circumstances that incur the violence, response and reporting method).

The Primary Care Posttraumatic Stress Disorder (PC-PTSD) developed by Prince was used to screen nursing students for PTSD after experiencing violence [19]. There were four items in the PC-PTSD: Have had nightmares about it or thought about it when you did not want to? Tried hard not to think about it or went out of your way to avoid situations that reminded you of it? Were constantly on guard, watchful, or easily startled? Felt numb or detached from others, activities, or your surroundings? Each item is marked by ‘yes’ or ‘no’. Weighted kappa coefficients were calculated which represent the quality of efficiency (k(0.5)), sensitivity (k(1)), and specificity (k(0)). The PC-PTSD scale had an optimally efficient cutoff score of 3 (k(0.5) = 0.61), with a sensitivity rate of 0.78, a specificity rate of 0.87, a positive predictive value of 0.65, and a negative predictive value of 0.92.

The Professional Identity Questionnaire for Nursing Students (PIQNS) was developed by Hao [20]. The PIQNS included 17 items and five dimensions: professional self-image, benefit of retention and risk of turnover, social comparison and self-reflection, independence of career choice, and social modelling. The items were rated on a 5-point scale. Higher score indicates higher professional identity. This scale is widely used in China. The Cronbach’s α of the PIQNS was 0.827 and the split-half reliability was 0.842. Five factors explained 58.88% of the total explained variance through the method of exploratory factor analysis. The reliability and validity of the scale were good.

### Data analysis

Data analysis was conducted with SPSS 24.0. Descriptive statistics were used to analyse the demographic characteristics of the participants and the current situation of WPV upon the nursing students. A chi-square test was used to examine the differences in the occurrence of WPV among demographic characteristics. Hierarchical regression analysis was applied to explore the relationship between the experience of WPV and professional identity. An alpha level of 0.05 was used for all statistical tests. The missing values of general information and professional identity of nursing students were replaced by median.

## Results

### Demographic characteristics

Table 1 reveals that 954 nursing students participated in the survey. 89.31% were female, 93.08% aged between 18 and 23 years old. Most of them stayed in the internship for more than 6 months. 61.22% were studying for junior college degree and others were studying for bachelor’s degree or higher. Only 33.54% had attended the violence prevention training. More than half of the students reported the concern about the violence during the internship (Table 1).

**Table 1:** Participants’ demographic characteristics (*N* = 954)

Variables	N	%
Gender		
Female	852	89.31
Male	102	10.69
Age (year)		
18–20	68	7.13
21–23	820	85.95
≥ 24	66	6.92
Place of residence		
City	491	51.47
Rural area	463	48.53
Are you the only one child in the family?		
Yes	539	56.5
No	415	43.5
Education level		
Bachelor’s degree or higher	370	38.78
Junior college	584	61.22
School location		
Shanghai	639	66.98
Suzhou	24	2.52
Dali	76	7.97
Nanchang	71	7.44
Hefei	144	15.09
Duration of the current internship		
≤ 6	10	1.04
>6	944	98.95
Student leader		
Yes	314	32.91
No	640	67.09
Have you joined school clubs?		
Yes	667	69.92
No	287	30.08
The reason for choosing nursing as your college major		
Voluntary application	539	56.5
Family member’s suggestion	219	22.96
Major change	196	20.55
Attended the violence prevention training?		
Yes	320	33.54
No	643	66.46
Concern about the violence during the internship		
Not at all	58	6.08
Not too much	261	27.36
Not sure	110	11.53
Slightly worried	435	45.6
Extremely worried	90	9.43

### Types of WPV

Among the 954 nursing students of this study, 42.98% experienced at least one case of violence in the past year during internship. The types of violent incidents ranging from high to low were: verbal abuse (38.47%), threat (14.78%), physical attack (2.73%), sexual harassment (1.99%), and gathering disturbance (medical dispute) (1.78%) (Table 2).

**Table 2:** Incidence of WPV among nursing students

Types of WPV	N	%
Verbal abuse	367	38.47
Threat	141	14.78
Physical attack	26	2.73
Sexual Harassment	19	1.99
Gathering Disturbance	17	1.78
Total	410	42.98

### Comparison of the occurrence of WPV by different characteristics

It was disclosed in the study that the WPV incidents differ statistically in various education levels, school location, major selection incentives and degree of concern about violence. The violence incident rate was higher in the students with bachelor’s degrees than with junior college degrees. The violence incident rate was high in the students who had changed their major in the college and those highly concerns about violence (Table 3).

**Table 3:** Occurrence of WPV by characteristics of the nursing students

Variables	Group without violence	Group with violence	Occurrence (%)	χ2	*P*
Gender				0.031	0.859
Female	485	367	43.08		
Male	59	43	42.16		
Age (year)				0.238	0.888
18–20	40	28	41.18		
21–23	465	355	43.29		
≥ 24	39	27	40.91		
Place of residence				1.382	0.240
City	271	220	44.81		
Village	273	190	41.04		
Single-child family				0.555	0.555
Yes	313	226	41.93		
No	231	184	44.34		
Education level				12.165	*P*<0.001
Bachelor’s degree or higher	185	185	50.00		
Junior college	359	225	38.53		
School location				16.543	0.002
Shanghai	388	251	39.28		
Suzhou	10	14	58.33		
Dali	39	37	48.68		
Nanchang	43	28	39.44		
Hefei	64	80	55.56		
Duration of the current internship				0.262	0.608^a^
≤ 6	7	3	30.00		
>6	537	407	43.11		
Student leader				0.082	0.775
Yes	177	137	43.63		
No	367	273	42.66		
Join school clubs				1.775	0.183
Yes	371	296	44.38		
No	173	114	39.72		
Major selection incentives				10.398	0.006
Voluntary application	329	210	38.96		
Family’s suggestion	121	98	44.75		
Major transfer	94	102	52.04		
Received training about violence prevention				0.393	0.531
Yes	187	133	41.56		
No	357	277	43.69		
Concern about violence during the internship				26.258	*P*<0.001
Not at all	40	18	31.03		
Not too much	172	89	34.10		
Not sure	69	41	37.27		
Slightly worried	225	210	48.28		
Extremely worried	38	52	57.78		

^a^Continuity (Yates) correction

### Characteristics of WPV among nursing students

The ranking of highest risk of WPV department – emergency department (38.05%), surgical department (11.12%), medical department (10.49), outpatient department (10.24%), etc. The perpetrators were mostly relatives of the patients (60.98%) and patients themselves (31.64%). Most WPV were caused by: perpetrators’ offensive physical behaviour (44.63%), dissatisfaction with nursing performance (38.29%), long time waiting (31.71%), requests rejected unreasonably (29.27%), etc. The responses of the nursing students to the WPV included: avoiding conflict (51.71%), explaining with patience (50.49%), seeking help from colleagues or teachers (31.95%), etc. 86.34% of the nursing students didn’t report the violence incidents. The reasons for the low incident reporting included being unaware of how to report (27.07%), indifference (20.49%), no response will happen (16.34%), etc. 11.22% of the nursing students consider WPV part of their job. 10.98% of the nursing students got 3 points of PT-PTSD score, 40.73% got 4 points (Table 4).

**Table 4:** Frequency of WPV toward nursing students by the characteristics

Variables	Frequency	
	N	%
Department		
Emergency department	156	38.05
Surgical department	46	11.22
Medical department	43	10.49
Outpatient department	42	10.24
Obstetrics and Gynaecology department	13	3.17
Orthopedics department	10	2.44
Intensive care unit	8	1.95
Psychiatry department	8	1.95
Pediatric department	7	1.71
Perpetrator		
Relatives of patient	250	60.98
Patient(s)	129	31.46
Registered nurse(s)	8	1.95
Doctor(s)	7	1.71
College internship	3	0.73
The circumstances that incur the violence		
Perpetrators’ offensive physical behaviour	183	44.63
Unsatisfied with the nurses’ performance	157	38.29
Long time waiting	130	31.71
Unreasonable requests were rejected	120	29.27
Unsatisfied with the treatment	93	22.68
Unsatisfied with the doctor’s work	75	18.29
Overestimation of the medical expenses	53	12.93
The patient died	46	11.22
Seeking financial compensation	39	9.51
Mental disorder	39	9.51
Drunken	38	9.27
Drug addiction	6	1.46
Response		
Avoid conflict	212	51.71
Explain with patience	207	50.49
Seek help from colleagues or teachers	131	31.95
Seek help from security guard	111	27.07
Seek help from chief nurse	70	17.07
Seek help from the police	53	12.93
Reason before fighting back	32	7.80
Seek help from other patients and family members	9	2.20
Conflict with each other (eg. Shouting/clashing)	8	1.95
Release a report?		
No	354	86.34
Reasons for not releasing a report		
Unaware of how to report	111	27.07
Indifference	84	20.49
No response will happen	67	16.34
It’s part of the job	46	11.22
I am afraid I will be revenged	13	3.17
Yes	37	9.02
PT-PTSD (score)		
1	72	17.56
2	62	15.12
3	45	10.98
4	167	40.73

### Relationship between the experience of WPV and professional identity

The differences of all dimensions and total score of the PIQNS between groups with or without WPV have statistical significance (*P* < 0.05), except the “social comparison and self-reflection”. The nursing students without WPV experience scored higher in professional identity than those with WPV (Table 5).

**Table 5:** Analysis of independent-samples *t*-test

	Group with violence	Group without violence	*t*	P
Professional self-image	20.67 ± 5.08	21.93 ± 4.76	3.924	<0.001
The benefit of retention and Risk of conflict	13.41 ± 3.40	14.26 ± 3.25	3.907	<0.001
Social comparison and self-evaluation	11.27 ± 1.96	11.44 ± 1.98	1.351	0.177
Independence of career choice	6.85 ± 1.48	7.17 ± 1.50	2.236	0.001
Social modelling	7.44 ± 1.81	7.72 ± 1.56	2.507	0.012
Total points	59.45 ± 11.14	61.98 ± 10.72	3.548	<0.001

### Results of hierarchical linear regression analysis

Firstly, independent-samples t-test and analysis of variance were used to compare the professional identity of nursing students with different characteristics. The results showed that there were differences in professional identity among students with different education levels, different major selection incentives and different situations of receiving training in violence prevention (*P*<0.05). The indexes with statistically significant differences in univariate analysis were put into the multivariate stratified regression model as covariates.

Variance inflation factor (VIF) was used to identify multicollinearity for each model. Both multivariate models waived the risk of collinearity (VIF > 10). VIF in each model was smaller than 2, and the variance inflation factors of the six influencing factors of the final model are between 1.015 and 1.423. Our hierarchical linear regression analysis results (Table 6) show that WPV experience (β = − 0.076, *P* < 0.05) is a significantly negative predictor of professional identity in step 2 (Table 6).

**Table 6:** Hierarchical Linear Regression Analysis Results

Variable	Professional Identity			
	Step1		Step2	
	*β*	*P*	*β*	*P*
**Block 1**				
Education level	0.163	<0.001	0.156	<0.001
Major selection incentives				
Family’s suggestion	−0.263	<0.001	−0.259	<0.001
Major transfer	−0.151	<0.001	−0.147	<0.001
Voluntary application (reference)	0.000	<0.001	0.000	<0.001
Received training about violence prevention	−0.123	<0.001	0.121	<0.001
**Block 2**				
WPV experience			−0.076	0.014
***F***	31.805	<0.001	26.789	<0.001
***R***^***2***^	0.119		0.124	
***△R***^***2***^	0.119		0.006	

## Discussion

In this study, 42.98% of the 954 nursing students had experienced at least one case of violence in the past year during their internship. Among the various types of violence, the prevalence of nonphysical violence (verbal abuse 38.47% and bullying 14.78%) was much higher than those of physical violence (physical attack 2.73%, sexual harassment 1.99% and gathering disturbance 1.78%). Similar patterns were observed in regional and global statistics data. Spector et al. [21] indicated that non-physical violence (65.5%) was higher than physical violence (26.7%) globally. The gap between the finding of Spector et al. [21] and this study may relate to differences in health care environments, culture, and perception or definition of violence across different people and cultures. Considering the prevalence of global WPV reported by qualified staff, it is not surprising that such a significant number of nursing students experiencing similar events. The unacceptable circumstances should raise attention and awareness of WPV among nursing students. Adequate training in identifying, reporting and management of WPV should be introduced in the school and the clinical provider.

The second valuable finding of this study was to identify high-risk groups of nursing students. As mentioned before, senior degree nursing students encountered more violence than junior degree holders. Students who had changed their major and those who concerned more about violence encountered more violence. Consistent with previous literature, nurses with university degrees, higher workload or stress, worse adaption to the environment may get higher risk of experiencing any form of violence [22, 23]. This study found that the main responses of nursing students to the WPV were avoiding conflict and explaining with patience. Few students sought help from teachers, security guards, and police. In the future, more efforts should be placed on building students’ prevention and response-ability in the training programs.

Thirdly, the nursing students were main target of hospital violence. The emergency department was one of the high violence targets in the hospital. In the emergency department, high-risk patients, such as patients experiencing an episode of mental illness or inebriated patients, and longer waiting time, were determined to be precipitating factors of potential violent behaviour [24]. High patient expectations [25], and the negative propaganda in the media [26] lead to patients aggressive behaviour. Patients, for instance, took for granted that they deserve high-quality care and good clinical outcomes once they are admitted to the hospital, regardless of the severity of their disease [27]. However, these accusations are frequently one-sided presenting only the patient’s point of view and are inaccurate, thus creating public distrust and anger toward medical professionals [26]. Something always happens at the beginning of the violence, such as offensive behaviour, unsatisfied with nurses’ performance, rejected unreasonable requests, etc. It is important to develop the ability of early identification, assessment, reporting and management of WPV among the nursing students.

Fourthly, the impact of WPV on individuals, in terms of mental well-being, should not be underestimated, since students often need additional support to cope with and manage challenging situations. Perhaps the most alert finding in this study was that 86.34% of the nursing students didn’t report violence incidents. The reasons include: unaware of how to report (27.07%), indifference (20.49%), no response will happen (16.34%), etc. 11.22% of the nursing students considered WPV as part of their jobs. The culture which tolerates the violence incidents in clinical practice is unacceptable and should be abandoned. It was disclosed that students or novice nurses might come up against high rates of negative behaviour during their time in practice [28]. Laschinger [29] suggested negative work experiences may result in new graduates assimilating such behaviour and displaying the same toward others. It was found in this survey that more than half of the nursing students developed post-traumatic stress disorder after WPV, which suggested clinical nursing teachers should support the students in psychological adjustment and recovery after the occurrence of violence.

The adverse effects of violence on nursing students’ mental health and professional identity have been revealed. Students should start raising awareness when preparing for their clinical placements. The process should include information to help understand and identify WPV and get access to clear information on how to report incidents. Students should be confident that incidents have to be handled properly with post-incident support through counselling and debriefing. Schools and placement providers should also provide training to mentors to assess the learning environment through audit and post-placement evaluation and provide debriefing sessions to students to accumulate their experience. Cooperation between academics in situations and service providers is critical to create best learning environments for students and build capacity for tomorrow’s workforce [30]. Nurse education institutions and health service providers should work together to better protect the nursing students and develop shared policies and procedures which raise understanding and awareness of the consequences and management of bullying/harassment [31], build up a culture of zero tolerance toward such behaviour and set up a self-valued society.

Nursing educators and administrators should focus on the population of nursing students who are vulnerable to violence. Targeted violence prevention training sessions can be developed in conjunction with the violence occurrence characteristics and nursing student coping weaknesses identified in this study. In addition, hospitals could have some psychological counselors to help nursing students after they have experienced violence.

Clinical nursing teachers need to be aware of the impact of violence on the professional identity of nursing students. Close attention should be paid to psychological experience and changes in professional values of nursing students. Nursing educators could identify and adjust the negative mindset of nursing students early by establishing peer support system to guide them in a healthy and positive direction.

### Limitations of the study

Firstly, this study was conducted in five cities in China by the convenience sampling, which affected the representativeness of samples to some extent, and the results may not apply to other regions. Secondly, whether the occurrence of WPV, coping style, and professional identity of nursing students have changed before and after clinical practice needs longitudinal research. In the future, it will also be necessary to deeply explore nursing students’ understanding and experience of violence through qualitative research.

## Conclusion

The results of this study demonstrated that nursing students were exposed to WPV during clinical practice, including physical and psychological behaviour. In addition, coping solutions and psychological adjustment of nursing students to violence were not satisfactory. The experience of violence significantly worsened the professional identity of nursing students, which harmed the quality of care and affect the long-term development of nursing. These results also highlighted the importance of violence prevention education during professional learning, especially training on risk assessment, how to cope with WPV, reporting WPV exposure and psychological recovery. More efforts are needed to reduce and prevent violence in the future.

## Data Availability

The datasets used and analysed during the current study are available from the corresponding author on reasonable request.

## Abbreviations

WPV: Workplace violence
STROBE: Strengthening the Reporting of Observational Studies in Epidemiolog
HWVQNS: Hospital Workplace Violence Questionnaire for Nursing Students
PC-PTSD: The Primary Care Posttraumatic Stress Disorder
PIQNS: The Professional Identity Questionnaire for Nursing Students
VIF: Variance inflation factor
