# Correction to: Characteristics of workplace violence, responses and their relationship with the professional identity among nursing students in China: a multicenter cross-sectional study

**DOI:** 10.1186/s12912-022-01052-4

**Published:** 2022-10-10

**Authors:** Lingyan Zhu, Dongyan Lu, Zhenlan Luo, Mengqi Xu, Linfang Sun, Sanlian Hu

**Affiliations:** 1 Department of Nursing, Shanghai JiaoTong University Affiliated Sixth People’s Hospital, Shanghai, China; 2 School of Nursing, Huzhou University, Huzhou, China; 3 School of Nursing, Shanghai JiaoTong University School of Medicine, Shanghai, China

**Correction to**: *BMC Nursing* (2022) 21:262. 10.1186/s12912-022-01037-3.

Following publication of the original article [1], the authors identified an error in the author names. The given names and family names of three of the authors were erroneously transposed.

The incorrect author names are: Lu Dongyan, Xu Mengqi and Hu Sanlian.

The correct author names are: Dongyan Lu, Mengqi Xu and Sanlian Hu.

The author group has been updated above and the original article [1] has been corrected.
